# The role of neutrophil extracellular traps in cancer progression, metastasis and therapy

**DOI:** 10.1186/s40164-022-00345-3

**Published:** 2022-11-16

**Authors:** Yue Chen, Haoyue Hu, Songtao Tan, Qionglan Dong, Xue Fan, Yi Wang, Huan Zhang, Jun He

**Affiliations:** 1 Department of Pathology, Beijing Shijitan Hospital, Capital Medical University, Beijing, 100038 China; 2 Department of Medical Oncology, Sichuan Cancer Hospital and Institute, Sichuan Cancer Center, Medicine School of University of Electronic Science and Technology, Chengdu, 610041 China; 3 West China School of Medicine, Sichuan University, Chengdu, 610041 China; 4 Southeast Medical University, Luzhou, 646099 China; 5 Southern Medical University, Guangzhou, 510515 China; 6 Department of Oncology, The Third Hospital of Mianyang (Sichuan Mental Health Center), Mianyang, 621015 China

**Keywords:** Neutrophil, Neutrophil extracellular traps, Cancer, Metastasis, Targeted therapy

## Abstract

Neutrophil extracellular traps (NETs) released by activated neutrophils typically consist of DNA-histone complexes and granule proteins. NETs were originally identified as a host defense system against foreign pathogens and are strongly associated with autoimmune diseases. However, a novel and predominant role of NETs in cancer is emerging. Increasing evidence has confirmed that many stimuli can facilitate NET formation in an NADPH oxidase (NOX)-dependent/NOX-independent manner. In cancer, NETs have been linked to cancer progression, metastasis, and cancer-associated thrombosis. In this review, we aimed to summarize the current available knowledge regarding NET formation and focused on the role of NETs in cancer biological behaviors. The potential target for cancer therapy will be further discussed.

## Background

Cancer-related inflammation has long been recognized as a driving force of tumorigenesis development. Increasing evidence has shown that immune cells constitute the innate and adaptive immune system and enable the ability of tumor cells to escape immunosurveillance [1]. Neutrophils (innate immune cells) are the most abundant heterogeneous leukocytes in humans, and play a critical role in host defense against pathogens, including bacteria, fungi, and viruses. A number of mechanisms are involved, including reactive oxygen species (ROS) production, phagocytosis, and degranulation [2]. In 2004, Brinkmann et al. discovered a novel immune defense mechanism of neutrophils called neutrophil extracellular traps (NETs), a special form of degranulation [3]. NETs are composed of DNA fibers, histones, granular content, and antimicrobial proteins, which contribute to entrapping and killing invasive bacteria [4]. A unique type of cell death accompanied by the formation of NETs is known as neutrophil extracellular trap-osis (NETosis), which unlike apoptosis and necrosis, is dependent on the generation of ROS by NADPH oxidase [5]. Under relevant stimuli (microbial infection or foreign invasion), neutrophils are rapidly activated and accumulate, after which they undergo morphological changes. These events can be found in sequence in cells undergoing NETosis with nuclear envelope disintegration, mixing of nuclear and cytoplasmic material, cytoplasmic organelle disappearance, chromatin decondensation, cell membrane rupture, and NET release [6]. Apart from the primary advantageous role of protection against foreign bodies, and when neutrophils are dysregulated, NETs are implicated in many inflammatory diseases, including gout, rheumatoid arthritis, systemic lupus erythematosus, and others [7]. In the last two decades, several studies focused on investigating the role of NETs in malignant tumors because of their vital roles in infectious and immune-related diseases [8]. On the one hand, NETs can exert an antitumor effect by directly killing tumor cells and inhibiting tumor growth and metastasis. On the other hand, NETs have been shown to contribute to exerting protumor activity by inhibiting apoptosis and inducing tumor angiogenesis. Although many studies have shown that NETs may be more inclined to promote tumor proliferation and metastasis, their role has not yet been completely elucidated [9, 10].

Furthermore, interactions between neutrophils and other immune cells in the tumor microenvironment (TME) have been demonstrated in previous studies. However, the underlying mechanism of interaction between these cells in the TME is still inconclusive [11]. Studies in this field have become the focus and difficulty of current cancer research, which will bring an understanding of identifying cancer biomarkers and developing novel therapeutics. Until now, NETs have been found in animal models, peripheral blood, and tumor specimens from cancer patients. In this review, we will focus on the known steps of the biological characteristics of NETs, tumor progression, and metastasis, and cancer-associated thrombosis. Subsequently, the role of potential NET markers as prognostic biomarkers and their ability to serve as potential targets for cancer therapy will be discussed.

## Mechanism of NET formation

In general, neutrophils are recognized as the core component of the innate immune system and play roles in endothelial adherence, chemotaxis, oxidization, phagocytosis, and the release of toxic granules, resulting in microbial killing [12]. The formation of NETs widely exists in neutrophils, which are considered a defense mechanism in response to harmful stimuli (bacteria, fungal hyphae, immune complexes, activated platelets, and biochemical stimuli) [7, 13]. NETs are mainly composed of DNA filaments with a diameter of 15–17 nm and many spherical protein substances with a diameter of approximately 25 nm. NET proteins have been identified as histones H1-H4, neutrophil elastase (NE), myeloperoxidase (MPO) and cathepsin G. Among NET proteins, histones H2-H4 account for approximately 70% and are core proteins [14]. In previous studies, it was widely believed that the formation of NETs is presumably a biologically conserved process. Two key mechanisms of NET formation have been discovered based on the death fate of neutrophils (NADPH oxidase-dependent/independent or NOX-dependent/independent suicidal NET formation) [15]. However, the underlying molecular mechanisms of NET formation are still not entirely understood.

### NADPH oxidase-dependent NET formation

**Fig. 1:**
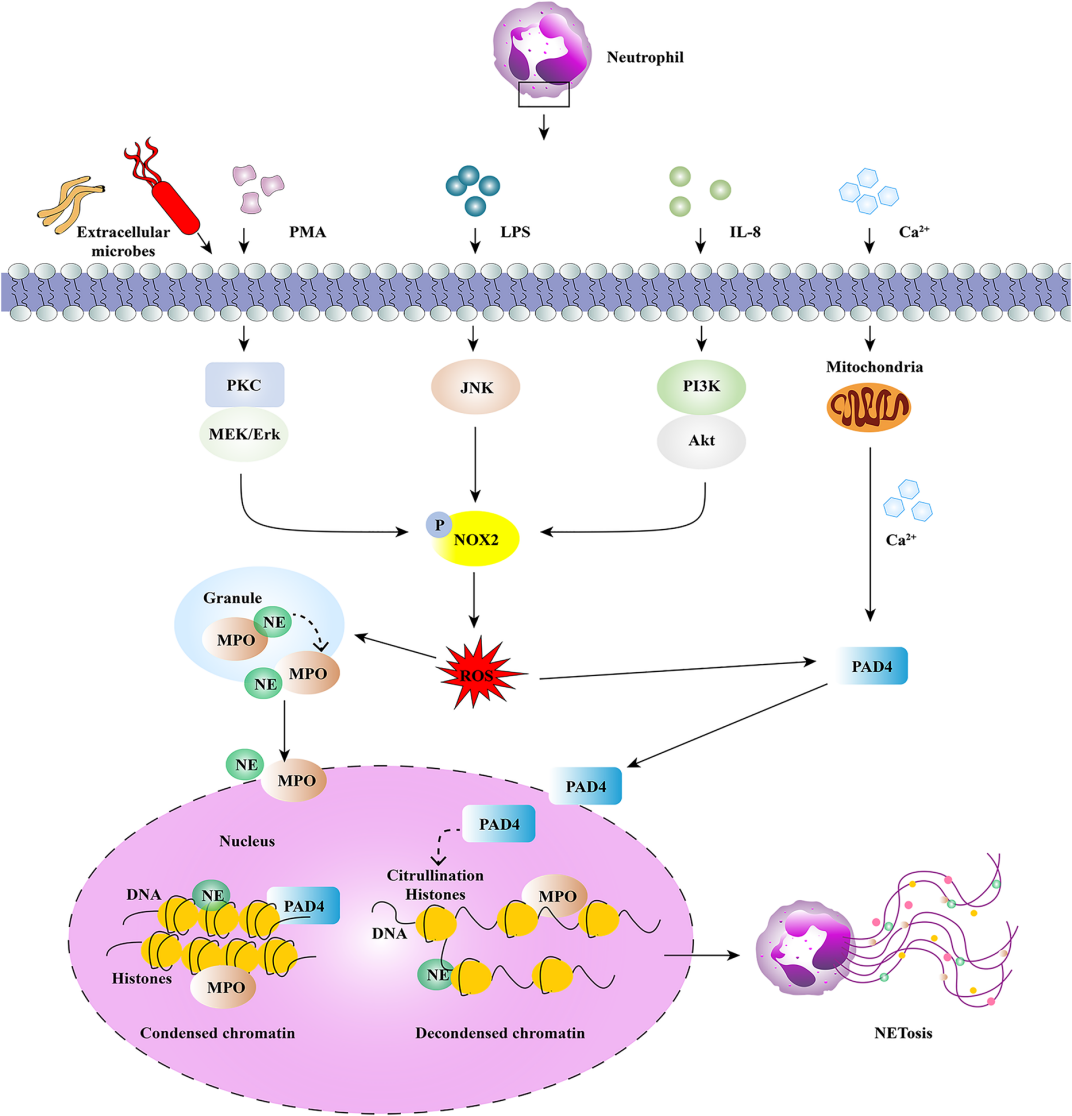
NADPH oxidase (NOX)-dependent NET formation pathways. Neutrophils are activated by extracellular microbes or PMA, LPS and IL-8, followed by activation of various pathways, including MEK/Erk, c-JNK, and PI3K/Akt signals. The endoplasmic calcium in the cytoplasm then phosphorylates NADPH Oxidase (Nox2), thereby driving the production of reactive oxygen species (ROS). Subsequently, neutrophil elastase (NE) and myeloperoxidase (MPO) stored in cytoplasmic granules translocate into the nucleus and contribute to chromatin decondensation with the assistance of calcium-dependent protein protein-arginine deiminase type 4 (PAD4), which citrullinates histones. Decondensed chromatin mixed with granule proteins is first released into the cytoplasm and then out of the cell membrane, and forms Neutrophil Extracellular Traps (NETs).

Under physiological conditions, histones are highly wrapped around most DNA strands in the nuclei of neutrophils, which results in transcriptional inactivity because of the constraint of DNA extension by protein‒DNA interactions. Under various stimuli conditions, such as pathogenic microorganisms in vivo or phorbol 12-myristate 13-acetate (PMA), interleukin-8 (IL-8), and lipopolysaccharide (LPS) in vitro, neutrophils can be activated [16, 17]. After 3 to 8 h of neutrophil activation, NADPH oxidase (NOX)-dependent NETs will form, which is the first discovered mechanism of NET formation. It is a type of suicidal approach because the formation of NETs is followed by neutrophil death [15]. Different from apoptosis and necrosis, this unique type of neutrophil death is considered NETosis, which is insensitive to caspase inhibition. In this period, neutrophils experience a series of biochemical and biological processes, which eventually lead to the release of potential energy along with chromatin and granulated protein expulsion [18]. The vital step during NETosis is the decondensed DNA strands into fibrous polymers in neutrophils. The critical step of chromatin decondensation is the generation of ROS, which is mediated by NADPH oxidase 2 (Nox2) [7]. Following PMA stimulation, the activity of protein kinase C was increased for the entry of endoplasmic calcium into the cytoplasm, which then phosphorylates Nox2, thereby driving the production of ROS [19]. Similar to PMA, phosphorylation of Nox2 was followed by LPS stimulation through the c-Jun N-terminal kinase (JNK) pathway [20]. Two key protein enzymes, MPO and NE, which are stored in the cytoplasmic granules of naïve neutrophils, can be released and activated by ROS. Once NE is translocated into the nucleus, chromatin decondensation starts. First, core histones (H2A, H2B, H3, and H4) that package nuclear DNA were disrupted and degraded by activated NE. Subsequently, MPO binds to nuclear chromatin for further decondensation with NE [21]. In addition to NE and MPO, another vital protease, peptidyl-arginine deiminase-4 (PAD4), catalyzes the conversion of arginine to citrullines in histones. In this process, citrullinated histones strongly weaken histone-DNA binding, which further boosts the chromatin decondensation of nuclear DNA [22]. Previous studies have demonstrated that PAD4 or NE deficiency affects NET formation in mouse models [23, 24]. However, the role of PAD4 remains controversial because in various studies, it has been shown that PAD4 is not always essential for NET formation [25]. After breakdown of the nuclear membrane, decondensed chromatin decorated with histones is released into the cytoplasm, mixes with granule proteins and extrudes throughout the cellular membrane after disintegration of the plasma membrane with the help of gasdermin D, which results in the release of NETs and neutrophil death [26, 27] (Fig. 1 and Table 1).

**Table 1:** Tumor-derived factors in recruiting neutrophils and inducers of NETs formation

Stimulus	Receptor	Function	Cancer type	References
Cytokines				
CXCL5	CXCR2	Promote cancer growth and metastasis by TAN chemotaxis and EMT induction	HCC, LC	[28, 29]
IL-8	CXCR1/2	Promote tumor progression and metastasis by TAN chemotaxis and activation	HCC, LC, CRC, Melanoma	[30–32]
IL-6	CXCR1/2	Promote cancer growth and metastasis by TAN chemotaxis	HCC, GC	[33, 34]
IL-17	IL-17R	Promote cancer growth and metastasis by TAN chemotaxis and activation	HCC, CRC, BC	[35–37]
IL-1β	-	Promote tumor progression and metastasis	CRC	[38]
G-CSF	G-CSFR	Promote cancer growth and metastasis by TAN chemotaxis	LC, BC	[39, 40]
HMGB1	TLR4, RAGE	Promote tumor angiogenesis and metastasis by TAN chemotaxis and NETs formation	HCC, CRC, Melanoma	[41, 42]
TGF-β	TGF-βR	Promote cancer growth by neutrophils N2 phenotype transformation	LC, BC	[43, 44]
Chemical stimulation				
PMA	PKC	Promote cancer growth and metastasis by NETs formation	LC, BC, PDAC	[19, 45, 46]
LPS	TLR4	Promote cancer growth and metastasis by NETs formation	CRC, LC, BC	[20, 45, 47]
Ca^2+^	-	Trigger NETs formation	-	[19, 22, 48]
Extracellular microbes				
Bacteria, Viruses, Fungi	-	Trigger NETs formation	-	[49–51]

*NETs* Neutrophil extracellular traps; *HCC* Hepatocellular carcinoma; *LC* Lung cancer; *BC* Breast cancer; *CRC* Colorectal cancer; *PDAC* Pancreatic ductal adenocarcinoma; *CXCL* C-X-C motif chemokine ligand; *CXCR* C-X-C motif chemokine receptor; *IL* Interleukin; *G-CSF* Granulocyte colony stimulating factor; *G-CSFR* Granulocyte colony stimulating factor receptor; *HMGB1* High mobility group box 1; *TLR* Toll-like receptor; *RAGE* Receptor for advanced glycation endproducts; *TGF-β* Transforming growth factor-β; *PMA* Phorbol 12-myristate 13-acetate; *PKC* Protein kinase C; *LPS* Lipopolysaccharide

### NADPH oxidase-independent NET formation

In 2004, in the first few years since NETs were first identified, the term “NETosis” has been widely used. However, in 2018, it was strongly recommended that the term NETosis should be replaced with NETs because a great number of studies reported that the formation of NETs does not accompany neutrophil death [52, 53]. In fact, NADPH oxidase (Nox)-independent NET formation was described in 2012. This is a fast calcium-activated pathway. In recent years, the detailed molecular mechanisms related to it, have received great attention, and some breakthroughs have been made [54]. Although NADPH oxidase is not necessary for NET formation, ROS generation is required. Similar to PMA or LPS, some Nox-independent NET formation agonists, such as calcium ionophores, nicotine and A23187, have been suggested to trigger NETosis via mitochondrial ROS (mROS) that is generated by the activation of the calcium-activated small conductance potassium (SK) channel member SK3 [19, 48]. In the past few years, mROS production mediated by CK channels has been considered to be associated with apoptosis, but this process has been linked to several autoimmune diseases. Therefore, we propose a novel role for mitochondria in neutrophils as ROS generators to participate in Nox-independent NET formation, thereby playing a role in innate immune function [55, 56].

## NETs and cancer

Neutrophils play a vital role in cancer. Acting as an arm of neutrophils, NETs in cancer were first identified by Demers et al. Based on the status of the immune system or TME, the role of NETs is variable [57]. On the one hand, NETs play an antitumor role in colorectal cancer (CRC) and head and neck squamous cell carcinoma by inducing apoptosis [58, 59]. Recently, in some studies, it was reported that NETs may have an antitumor function in ovarian cancer and melanoma by inducing necrosis[60, 61]. The most direct mechanism may be its direct killing of tumor cells or stimulation of the immune system to fight against the tumor [62]. In melanoma, MPO is a representative component of NETs, which can kill melanoma cells (cell line A375) and decline the ability of proliferation and metastasis after implementation [61]. Higher values of the S100A8/CRP ratio, the release of which is associated with NETs, were found to correlate with favorable survival of high grade serous ovarian cancer (HGSOC) patients [60]. Moreover, neutrophils secrete high levels of H_2_O_2_ when stimulated by PMA that could inhibit metastatic seeding in the mouse lung cancer models [63]. On the other hand, an increasing number of studies have focused on the protumor role of NETs in various types of malignant tumors [lung cancer (LC), breast cancer (BC), and myeloproliferative neoplasms] through the promotion of tumor proliferation and metastasis [64–66]. Furthermore, NETs are associated with tumor angiogenesis and cancer-associated thrombosis [57]. Subsequently, the underlying mechanisms of NETs in tumor proliferation, metastasis, angiogenesis, and cancer-associated thrombosis will be highlighted.

### NETs and the tumor microenvironment

Neutrophils are leukocytes originating from the bone marrow and spleen and are considered the first line of defense against microorganism infections or injuries. Neutrophils are normally generated every day and can be further increased by proinflammatory factors during infection. NETs are also induced [67]. In recent years, in various studies, it has been demonstrated that NETs can also be stimulated by tumor cells in the absence of an infection and can act as important components of the TME, thereby playing a pivotal role in cancer [7]. Based on their function, there are two phenotypes of tumor-associated neutrophils (TANs) in the TME: the N1 (antitumor) phenotype and the N2 (protumor) phenotype [43]. N1 TANs have been demonstrated to enhance proinflammatory cytokines, including tumor necrosis factor-α (TNF-α) and intercellular adhesion molecule-1 (ICAM-1). On the other hand, C-X-C motif chemokine ligand 8 (CXCL8, also called interleukin-8, IL-8) and CXCL5 are upregulated in N2 TANs. In addition, N2 TANs in the TME are also associated with tumor angiogenesis by recruiting matrix metalloproteinase-9 (MMP-9) [68]. The upregulation of the expression of proinflammatory cytokines [including TNF-α, IL-8, and interleukin-6 (IL-6)] and neutrophil survival have been reported to promote protumorigenic (N2) phenotype TANs in a breast cancer model. In a lung cancer cell model, Shaul et al. showed that the immunosuppressive cytokine transforming growth factor-β (TGF-β) can polarize neutrophils into an N2 phenotype [69]. Granulocyte colony stimulating factor (G-CSF) can stimulate neutrophil production and maintain neutrophil survival in the bone marrow. The formation of NETs can be increased by the upregulation of G-CSF in the TME. As the upstream regulatory cytokine of G-CSF, interleukin-1 beta (IL-1β) was found to influence NET production in breast cancer [70]. Tumor-derived G-CSF can drive NET generation, and cancer-associated fibroblasts (CAFs) in the TME have recently been considered one of the key factors in NET formation [71]. Furthermore, the generation of IL-8 is mainly regulated by the upstream transcription factor nuclear factor kappa B (NF-κB). Interactions with neutrophils of IL-8 are exerted through C-X-C motif chemokine receptors 1 and 2 (CXCR1 and CXCR2). Tumor-derived IL-8 has been shown to induce NET generation in many malignant tumors, including BC, LC, hepatocellular carcinoma (HCC), and melanoma [72, 73]. From the above discussion, these findings demonstrate that NET formation is closely related to the TME and promotes pro-tumoral function (Table 1).

### NETs promote proliferation

Recent evidence has confirmed that neutrophils are a significant component of the TME, and a high neutrophil infiltration or a higher neutrophil to lymphocyte ratio is associated with faster progression and poor prognosis in various malignant tumors [74]. NETs play a complex role in tumor progression (proliferation and growth) by different mechanisms. First, in nonsolid tumors, such as chronic lymphocytic leukemia and diffuse large B-cell lymphoma (DLBCL), NETs have been shown to enhance the proliferative ability by increasing activation markers and inhibiting apoptosis of tumor cells [75]. Moreover, activation of the NF-κB pathway and signal transducer and activator of transcription 3 (STAT3)/p38 signaling stimulated by NETs is another proposed mechanism of promoting tumor proliferation in DLBCL [76]. Second, in solid tumors, multiple underlying mechanisms have been elucidated. Circulating tumor cells (CTCs) are cancer cells that “have fallen off” a tumor to circulate in the bloodstream and undergo a state of dormancy when exposed to an adverse microenvironment (lack of adequate angiogenesis and nutrient supply) [77]. In a mouse model injected with dormant MCF-7 BC cells, Albruenges et al. reported that NET formation and awakened tumor cell proliferation increased in sustained inflammatory lungs after exposure to LPS [45]. Multiple cytokines and chemokines secreted by tumor cells, including IL-8, IL-17, G-CSF, and CXCL6, can recruit bone marrow-derived neutrophils to tumor sites and trigger NETosis. Subsequently, there is increased neutrophil infiltration and NET formation in the TME, which ultimately leads to increased tumor cell proliferation [72, 78]. As a key component of NETs, NE plays an important role in the tumorigenesis of digestive system tumors (CRC and HCC). Yazdani et al. demonstrated that NETs can activate toll-like receptor 4 (TLR4)-proliferator activated receptor gamma coactivator 1-α (PGC-1α) signaling in CRC MC38 cells. Subsequently, mitochondrial adenosine triphosphate (ATP) is produced through the NE-activated TLR4-PGC-1α pathway, which is involved in tumor cell proliferation and metastasis [47]. In another MC38 CRC cell study, high mobility group box 1 (HMGB1), a constituent part of NETs, interacted with toll-like receptor 9 (TLR9), followed by activation of MAP kinase signaling to perform tumorigenic functions [79]. In addition, neutrophil infiltration and NETs formation were previously reported in neurological cancers. HMGB1, which acts as a ligand of RAGE, has been shown to participate in glioma tumor cell proliferation by activating the NF-κB pathway and promoting IL-8 secretion [80]. Furthermore, NETs protect tumor cells from cytotoxicity by suppressing infiltrating CD8 + and natural killer (NK) cells in the TME, thereby promoting tumor cell survival and growth [81].

### NETs promote metastasis

**Fig. 2:**
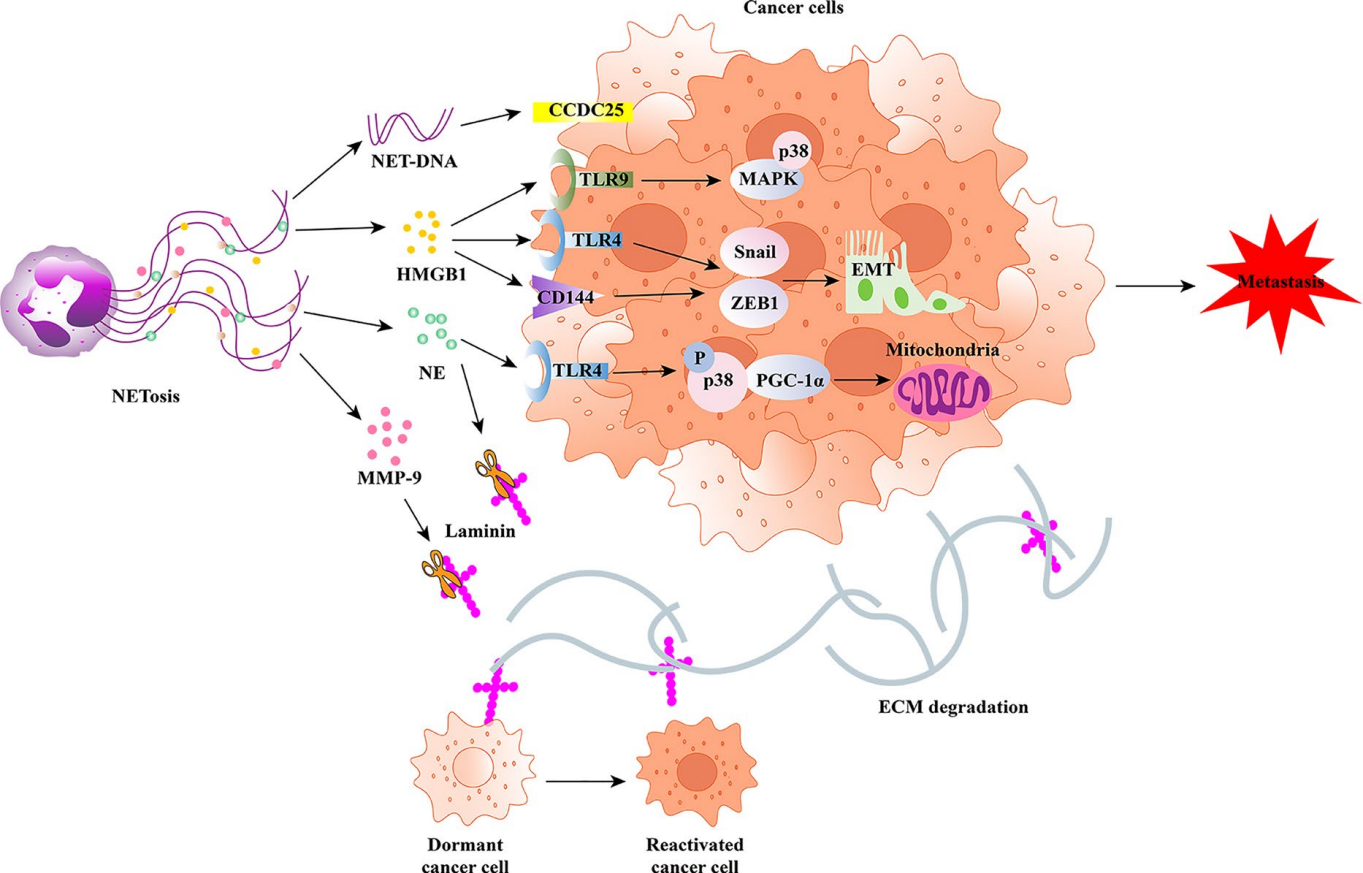
Molecular mechanisms of NETs in tumor metastasis. HMGB1, released by NETs, promotes tumor metastasis by binding to TLR9 to activate p38/MAPK signaling. HMGB1 also facilitates metastasis by binding to TLR4 or by regulating the degradation of VE-cadherin (CD144), followed by increased expression of EMT-associated genes, ZEB1 and Snail. The NETs component, NE, directly regulates mitochondrial metabolism via the TLR4-p38-PGC1-α pathway and promotes tumor proliferation and metastasis. NETs-derived NE and MMP-9 proteases are required for reactivating dormant cancer cells through degradation of the extracellular matrix (ECM) through the cleavage of laminin. In several cancer cells, NET-DNA directly binds to CCDC25, resulting in tumor metastasis.

Tumor metastasis, the process by which tumor cells spread from the primary lesion to a distant site (tissues or organs), is the leading cause of cancer-related death [82]. With a deeper understanding on the function of NETs in cancer, the relationship between NETs and cancer metastasis has become an emerging topic of interest. Recent studies have confirmed the underlying mechanisms of NETs in the tumor invasion-metastasis process. The epithelial to mesenchymal transition (EMT) is a crucial mechanism by which tumor cells acquire motility and invasiveness [83]. The degradation of VE-cadherin (CD144) accompanied by the activation of the Wnt/β-catenin pathway is an important process of EMT formation induced by NETs, which was first reported by Pieterse et al. [84]. NETs have been shown to change the morphology from MCF7 cells from an epithelial to a mesenchymal phenotype (EMT) in a BC mouse model, thereby promoting the tumor migration ability. The underlying mechanism of action involved upregulation of the expression of EMT-related genes, including ZEB1 and Snail [66]. Another in vitro study confirmed that the DNA component of NETs (NETs-DNA) significantly enhanced the migration and adhesion ability of BC cells (MDA-MB-231) with the help of coiled-coil domain containing protein 25 (CCDC25), which functions as a NET receptor and binds NETs-DNA [85]. Jin et al. revealed that the migration, invasion, and EMT in pancreatic ductal adenocarcinoma (PDAC) cells was promoted via IL-1b/epidermal growth factor receptor (EGFR)/extracellular signal regulated kinase (ERK) signaling when introduced to NET supernatant [46]. As mentioned earlier, CTCs undergo a state of dormancy under adverse conditions. However, dormant cells eventually break out of the dormant state and metastasize. Albrengues et al. demonstrated that NETs participate in the awakening of dormant tumor cells in mouse models of metastatic lung cancer. Lung inflammation stimulated by NETs (nasal instillation of LPS) can awaken dormant lung cancer cells and facilitate metastasis. Mechanistically, NET-derived NE and MMP-9 proteases were required for reactivating dormant cells through extracellular matrix (ECM) remodeling rather than through direct contact between NETs and dormant tumor cells. NE and MMP-9 are necessary to cleave and remodel laminin, which activates downstream integrin α3β1 and FAK/MEK/ERK signaling, subsequently allowing dormant tumor cells to reenter the cell cycle, leading to the resumption of aggressive metastatic growth of tumor cells [45]. In addition to its role in tumor proliferation, HMGB1 plays a nonnegligible role in tumor metastasis. HMGB1 is a NET-related component protein that can activate the TLR9 pathway and subsequently stimulate the p38 and JNK pathways to promote CRC cell migration and metastasis [79]. Furthermore, HMGB1 can enhance tumor migration and invasion abilities through EMT formation [83] (Fig. 2).

### NETs promote angiogenesis

Angiogenesis is a hallmark of malignant tumors and can provide sufficient oxygen and nutrients for tumor proliferation and metastasis [86]. The vascular endothelial growth factor (VEGF) family, including VEGF-A-D and placental growth factor (PlGF), are major angiogenic molecules [87]. VEGF levels were found to be higher in peripheral blood neutrophils of BC patients compared to that in neutrophils from healthy control [88]. Neutrophil-derived MMP-9 has been shown to be linked to VEGF activation and angiogenesis. In summary, neutrophils have higher levels of VEGF and MMP-9, which, in general, are connected to angiogenesis [36]. The angiopoietin (ANGPT) family includes ANGPT1 and ANGPT2, which are another group of key proangiogenic factors. ANGPT1 is produced by pericytes and some types of immune cells and acts as an agonist of the tyrosine kinase receptor TIE2 on endothelial cells (ECs). ANGPT2 is an antagonist of TIE2 [89]. Both ANGPT1 and ANGPT2 can induce neutrophil adhesion into ECs. Subsequently, NET formation increases by prolonging the incubation of neutrophils with ANGPT1/2 [90]. To investigate the relationship between NETs and angiogenesis, a series of in vivo experiments were conducted. Aldabbous and collaborators first revealed that NET-DNA can enhance vascularization by subcutaneous injection in a mouse model and that NETs can induce angiogenesis in human pulmonary artery endothelial cells (HPAECs) [90]. This evidence suggests that ANGPT1/2 can promote the formation of NETs, which exert proangiogenic functions.

### NETs and cancer-associated thrombosis

Thrombosis is caused by damage to ECs and blood clot formation, and blocks normal blood flow in arteries or veins. This pathological condition can lead to a variety of fatal diseases, including ischemic stroke and venous thromboembolism (VTE), which contribute to the global burden of disease [91]. Cancer-associated thrombosis has been identified as the second leading cause of death in cancer patients with hypercoagulable conditions. In previous studies, NETs have been found to contribute to thrombosis in infected wounds. In the last decade, NETs have significantly changed our view of cancer-associated thrombosis [92]. This may result from the interaction of various mechanisms. Recent data provide strong evidence that NETs enhance not only platelet adhesion, activation, and aggregation but also erythrocyte adhesion, which directly leads to fibrin deposition and clotting processes, thereby accelerating cancer-related thrombosis [93]. Functional release of platelets is directly caused by neutrophil-derived histone proteasomes in a TLR2- and TLR4-dependent manner [94]. In an in vivo model of pancreatic cancer, Norbaini et al. recently demonstrated that AsPC-1 pancreatic cancer cells can activate rapid NET formation. In addition, when platelets were incubated with neutrophils, they were preincubated with AsPC-1 cells, which can promote the release of NETs, thereby promoting thrombosis formation [95]. The same phenomenon was observed in gastric cancer patients, and NETs promoted the generation of thrombin and fibrin [96]. In myeloproliferative neoplasms (MPNs), NETs have been reported to contribute to thrombogenesis via platelet activation, which is a major cause of mortality in patients [97].

## NETs as potential therapeutic targets

Considering that NETs are closely related to a higher mortality and poor prognosis of cancer patients, inhibiting the related pathway of NET formation can be a potential therapeutic target to control cancer progression and metastasis. Given the above knowledge, blocking NET formation via small molecule drugs against NET constituents, such as DNase I [98, 99], NE inhibitors [100], MPO inhibitors [47], and PAD4 inhibitors [101, 102] may have great potential. As the core component of NETs, DNA can be targeted using DNase I. In an in vitro assay, DNase I treatment suppressed pancreatic cell growth and decreased gastric cancer cell adhesion [103]. Moreover, after DNase I treatment, gastric cancer cells exhibited an epithelial phenotype rather than a mesenchymal phenotype (invasive and migratory phenotype) [104]. In animal models, the tumor growth of human CRC and HCC was suppressed under DNase I treatment. Therefore, the metastatic potential of BC and LC was significantly reduced after DNase I therapy [105, 106]. Chromatin densification is the most critical step in NET formation and is dependent on the presence of PAD4 via histone citrullination. In global PAD4 gene-deleted mice, it was shown that NET production was decreased and tumor cell proliferation was mitigated compared to wild-type (WT) mice [47]. PAD4 deficiency can also promote tumor cell apoptosis and reduce metastatic burden [107]. Decades ago, the FDA approved DNase for cystic fibrosis patients, thereby demonstrating its safety profile as a drug [108]. Unfortunately, only a handful of clinical trials are being conducted to validate the effectiveness of DNase in cancer patients. Pulmozyme, a recombinant human DNase, is currently being tested in a phase 1 trial (NCT00536952) in patients with head and neck cancer who undergo radiotherapy and chemotherapy. In a phase 2 clinical trial in acute myeloid/lymphoid leukemia, Oshadi D and Oshadi R (DNase in an Oshadi carrier) were evaluated (NCT02462265). NETs have recently been linked to cancer resistance and immunotherapy. Previous studies have shown that deoxyribonuclease I (DNase I) disruption of NETs is promising in efforts to improve CAR-T efficiency [109]. In a CRC mouse model, light-regulated release of DNase I sensitized immune checkpoint inhibitors (ICIs) treatment [110].

**Fig. 3:**
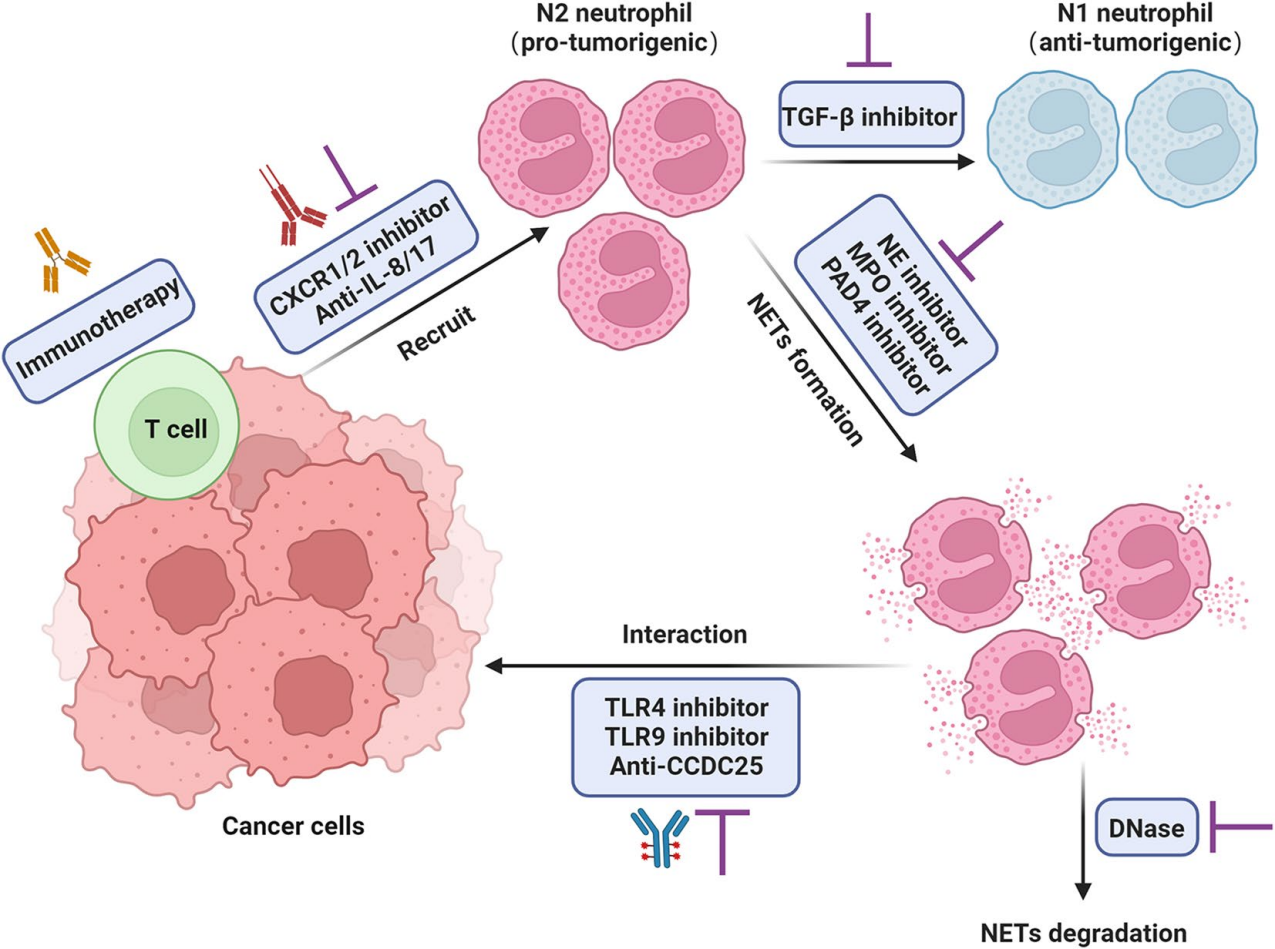
NETs as potential therapeutic targets. Inhibition of NETs formation via targeting crucial players, such as NE, MPO, or PAD4 in the formation pathway. Another way to inhibit NETs is by blocking CXCR1/2 and IL-8, key mediators of neutrophil chemotactic recruitment. Blockage of NETs-cancer cells interaction via targeting the TLR4/9 and CCDC25 can prevent the effect of NETs on cancer cells. As the core component of NETs, DNA can cause targeted destruction using DNAse. In addition, N2 neutrophils (pro-tumorigenic) can be converted to N1 neutrophils (anti-tumorigenic) by TGF-β inhibitors. Therapeutics used to target NETs may be a potential beneficial approach in combination with immunotherapy.

Another way to inhibit NETs is by blocking CXCR1 and CXCR2, which are key mediators of neutrophil chemotactic recruitment. The CXCR1/2-IL8 axis plays an important role in neutrophil chemotaxis as well as in NET formation [75]. Based on the protumor functions of the axis, CXCR1/2 and IL-8 have attracted much attention as therapeutic targets. IL-8 production may be triggered by IL-17, and inhibition of IL-17/IL-17RA signaling increases immune checkpoint blockade (anti-PD-1, anti-CTLA4) sensitivity in transplanted orthotopic PDAC mouse models [111]. Currently, in a phase1/2 clinical trial (NCT03400332) the combined treatment safety and efficacy of IL-8 inhibitor with nivolumab (anti-PD-1 mAb) or nivolumab plus ipilimumab (anti-CTLA4 mAb) is explored [112]. Several CXCR1/2 inhibitors combined with ICIs are currently being tested in clinical trials. SX-682, a CXCR1/2 inhibitor, is currently being tested in a phase 1 trial (NCT03161431) in advanced melanoma patients in combination with pembrolizumab (anti-PD-1 mAb). Navarixin, which targets both CXCR1/2, has been evaluated in combination with pembrolizumab in an ongoing phase 2 trial (NCT03473925) in advanced solid tumors. In addition, combination treatment with reparixin (another molecular drug against CXCR1/2) and paclitaxel showed antitumor activity along with a great safety profile in a phase 1b study in HER2-negative metastatic BC patients. A phase 2 clinical study is ongoing in patients with metastatic triple-negative BC (NCT02370238) [113] (Fig. 3). Table 2 summarizes potential therapeutics used to target NETs in cancer.

**Table 2:** Potential targets for anti-NETs in cancer

Target molecules	Mechanism	Agents	Cancer type	References
NETs components				
DNA	DNA Degradation	Recombinant DNase	CRC	[114, 115]
NE	NE inhibitor	GW311616	Leukemia	[116]
MPO	MPO inhibitor	PF-1355, ABAH, TX1	–	[54, 117]
PAD4	PAD4 inhibitor	GSK484, Cl-amidine	PDAC	[71, 118]
Chemokines				
CXCR1	CXCR1 inhibitor	SX-682, Reparixin	HNSCC, BC	[113, 119]
CXCR2	CXCR2 inhibitor	SX-682, Reparixin, SB225002, AZ13381758	HNSCC, BC, PDAC	[113, 119] [120, 121]
IL-8	Anti-IL-8	HuMab 10F8	–	[122]
IL-17	Anti-IL-17	IL17 blockade	PDAC	[111]
Transmembrane DNA receptor				
CCDC25	Anti-CCDC25	Anti-CCDC25 antibody	BC	[85]
Cytokines				
TLR4	TLR4 inhibitor	TAK-242	CRC	[123]
TLR9	TLR9 inhibitor	Hydroxychloroquine	–	[124]
TGF-β	TGF-β inhibitor	SB525334	PDAC	[125]

*NETs* Neutrophil extracellular traps; *HNSCC* Head and neck squamous cell carcinoma; *CRC* Colorectal cancer; *PDAC* Pancreatic ductal adenocarcinoma; *BC* Breast cancer; *NE* Neutrophil elastase; *MPO* Myeloperoxidase; *PAD4* Peptidyl-arginine deiminase-4; *CXCR* C-X-C motif chemokine receptor; *IL* Interleukin; *TLR* Toll-like receptor; *TGF-β* Transforming growth factor-β

These findings support the potential treatment of blocking NETs to effectively control tumor progression and metastasis. However, the current study has some limitations. Firstly, current studies on anti-NETs therapy mainly rely on xenograft mouse models, which do not reflect the complex microenvironment seen in tumor patients. Secondly, in current clinical trials, injection of these NET inhibitors may have off-target effects, especially in elderly cancer patients with compromised immunity. Finally, targeting NETs is quickly becoming an optimistic treatment option in the cancer field, but it is clear to see that unwanted effects on the immune system have been found [126].

## Conclusion

In recent years, increased attention has been given to tumor-associated neutrophils and their role in the TME. In addition, NETs from neutrophils play a complex and key role in cancer progression, metastasis, angiogenesis, cancer-associated thrombosis, and therapy. This review elaborates on the underlying mechanism of NET formation and its role in tumors. Most studies on the involvement of NETs in cancer biological behavior were based on animal or cellular models. Further studies are needed to understand the molecular mechanisms that regulate NET formation in tumors. Given their crucial roles in cancer, NETs have an important clinical application value. NET inhibitors against the components or receptors of NETs have great potential in the prevention and treatment of tumors. In previous studies, the combination of NET-interfering drugs and ICIs in the treatment of tumors has achieved some efficacy.

In conclusion, NETs will become possible therapeutic targets in cancer patients in the future, and clinical trials to verify the efficacy of NET-interfering drugs in cancers will be further explored.

## Data Availability

All data generated or analyzed during this study are included in this published article and its supplementary information files.

## Abbreviations

ATP: Adenosine triphosphate
BC: Breast cancer
CRC: Colorectal cancer
CAFs: Cancer-associated fibroblasts
CXCR1: C-X-C motif chemokine receptors 1
CXCR2: C-X-C motif chemokine receptors 2
CXCL8: C-X-C motif chemokine ligand 8
CXCL5: C-X-C motif chemokine ligand 5
CTCs: Circulating tumor cells
CCDC25: Coiled-coil domain containing protein 25
DLBCL: Diffuse large B-cell lymphoma
ECs: Endothelial cells
EMT: Epithelial to mesenchymal transition
EGFR: Epidermal growth factor receptor
ERK: Extracellular signal regulated kinase
ECM: Extracellular matrix
G-CSF: Granulocyte colony stimulating factor
G-CSFR: Granulocyte colony stimulating factor receptor
HGSOC: High grade serous ovarian cancer
HNSCC: Head and neck squamous cell carcinoma
HMGB1: High mobility group box 1
HPAECs: Human pulmonary artery endothelial cells
HCC: Hepatocellular carcinoma
IL-1β: Interleukin-1 beta
IL-6: Interleukin-6
IL-8: Interleukin-8
ICAM-1: Intercellular adhesion molecule-1
ICIs: Immune checkpoint inhibitors
JNK: C-Jun N-terminal kinase
LC: Lung cancer
LPS: Lipopolysaccharide
MPNs: Myeloproliferative neoplasms
MMP-9: Matrix metalloproteinase-9
MPO: Myeloperoxidase
NETs: Neutrophil extracellular traps
NOX: NADPH oxidase
NF-κB: Nuclear factor kappa B
NE: Neutrophil elastase
NK: Natural killer
PMA: Phorbol 12-myristate 13-acetate
PKC: Protein kinase C
PGC-1α: Proliferator activated receptor gamma coactivator 1-α
PDAC: Pancreatic ductal adenocarcinoma
PlGF: Placental growth factor
PAD4: Peptidyl-arginine deiminase-4
ROS: Reactive oxygen species
RAGE: Receptor for advanced glycation endproducts
STAT3: Signal transducer and activator of transcription 3
TME: Tumor microenvironment
TANs: Tumor-associated neutrophils
TNF-α: Tumor necrosis factor-α
TGF-β: Transforming growth factor-β
TLR4: Toll-like receptor 4
TLR9: Toll-like receptor 9
VEGF: Vascular endothelial growth factor
VTE: Venous thromboembolism
WT: Wild-type
